# Testing the Effectiveness and Cost-Effectiveness of a Combination HIV Prevention Intervention Among Young Cisgender Men Who Have Sex With Men and Transgender Women Who Sell or Exchange Sex in Thailand: Protocol for the Combination Prevention Effectiveness Study

**DOI:** 10.2196/15354

**Published:** 2020-01-27

**Authors:** Andrea L Wirtz, Brian Wilson Weir, Sandra Hsu Hnin Mon, Pachara Sirivongrangson, Tareerat Chemnasiri, Eileen F Dunne, Anchalee Varangrat, Andrew C Hickey, Michele R Decker, Stefan Baral, Kamolnetr Okanurak, Patrick Sullivan, Rachel Valencia, Michael C Thigpen, Timothy H Holtz, Philip A Mock, Betsy Cadwell, Adeola Adeyeye, James F Rooney, Chris Beyrer, Combination Prevention Effectiveness (COPE) Study Team

**Affiliations:** 1 Center for Public Health and Human Rights Department of Epidemiology Johns Hopkins Bloomberg School of Public Health Baltimore, MD United States; 2 Department of Health, Behavior & Society Johns Hopkins Bloomberg School of Public Health Baltimore, MD United States; 3 Department of Disease Control Thailand Ministry of Public Health Nonthaburi Thailand; 4 HIV/STD Research Program Thailand MOPH-US CDC Collaboration Nonthaburi Thailand; 5 Division of HIV/AIDS Prevention Centers for Disease Control and Prevention Atlanta, GA United States; 6 Department of Population, Family & Reproductive Health Johns Hopkins Bloomberg School of Public Health Baltimore, MD United States; 7 Faculty of Tropical Medicine Mahidol University Bangkok Thailand; 8 Department of Epidemiology Emory University Rollins School of Public Health Atlanta, GA United States; 9 Prevention Science Program Division of AIDS, National Institute of Allergy and Infectious Disease National Institute of Health Bethesda, MD United States; 10 Medical Affairs Gilead Sciences Foster City, CA United States

**Keywords:** HIV, prevention, pre-exposure prophylaxis, men who have sex with men, transgender persons, sex work, Thailand, cost-effectiveness

## Abstract

**Background:**

Pre-exposure prophylaxis (PrEP) is highly effective in the prevention of HIV acquisition, particularly for men who have sex with men (MSM). Questions remain on the benefits of PrEP and implementation strategies for those at occupational risk of HIV acquisition in sex work, as well as on methods to support adherence among young people who initiate PrEP.

**Objective:**

The Combination Prevention Effectiveness study for young cisgender MSM and transgender women (TGW) aims to assess the effectiveness and cost-effectiveness of a combination intervention among HIV-uninfected young MSM and TGW engaged in sex work in Thailand.

**Methods:**

This open-label, nonrandomized assessment compares the relative effectiveness of a combination prevention intervention with and without daily oral emtricitabine and tenofovir disoproxil fumarate (Truvada) PrEP with SMS-based adherence support. HIV-uninfected young MSM and TGW aged 18 to 26 years in Bangkok and Pattaya who self-report selling/exchanging sex at least once in the previous 12 months are recruited by convenience sampling and peer referral and are eligible regardless of their intent to initiate PrEP. At baseline, participants complete a standard assessment for PrEP eligibility and may initiate PrEP then or at any time during study participation. All participants complete a survey and HIV testing at baseline and every 3 months. Participants who initiate PrEP complete monthly pill pickups and may opt-in to SMS reminders. All participants are sent brief weekly SMS surveys to assess behavior with additional adherence questions for those who initiated PrEP. Adherence is defined as use of 4 or more pills within the last 7 days. The analytic plan uses a person-time approach to assess HIV incidence, comparing participant time on oral PrEP to participant time off oral PrEP for 12 to 24 months of follow-up, using a propensity score to control for confounders. Enrollment is based on the goal of observing 620 person-years (PY) on PrEP and 620 PY off PrEP.

**Results:**

As of February 2019, 445 participants (417 MSM and 28 TGW) have contributed approximately 168 PY with 95% (73/77) retention at 12 months. 74.2% (330/445) of enrolled participants initiated PrEP at baseline, contributing to 134 PY of PrEP adherence, 1 PY nonadherence, and 33 PY PrEP nonuse/noninitiation. Some social harms, predominantly related to unintentional participant disclosure of PrEP use and peer stigmatization of PrEP and HIV, have been identified.

**Conclusions:**

The majority of cisgender MSM and TGW who exchange sex and participate in this study are interested in PrEP, report taking sufficient PrEP, and stay on PrEP, though additional efforts are needed to address community misinformation and stigma. This novel multilevel, open-label study design and person-time approach will allow evaluation of the effectiveness and cost-effectiveness of combination prevention intervention in the contexts of both organized sex work and exchanged sex.

**International Registered Report Identifier (IRRID):**

RR1-10.2196/15354

## Introduction

### Background

Although there have been remarkable scientific advances in HIV prevention and control, many HIV epidemics globally remain concentrated among key populations [1-4]. In particular, men who have sex with men (MSM) and transgender women (TGW) continue to experience high HIV incidence [1,4]. Expanding HIV epidemics among MSM and TGW are occurring in high- and low-income countries, in North and South America and in South and Southeast Asia [1,5]. Though contextually different settings, the United States and Thailand have observed similar epidemiologic trends in HIV infections, with the highest rates of new HIV infections reported in the youngest age strata, among young MSM aged 15 to 24 years (in the United States) and among MSM aged 18 to 21 years in Thailand [1,6]. In a recent report from the United Nations Children's Fund Thailand, 55% of young MSM in Bangkok reported being sexually active at or below age 15 years, and young MSM accounted for 41% of all new HIV infections in the country in 2013 [7]. A 2005 venue-based study of TGW in 3 urban settings in Thailand found that 8.4% of TGW aged 15 to 22 years were already living with HIV and being aged 13 years or younger at the time of anal sexual debut was associated with a 2.5-fold increased odds of HIV infection [8]. Young MSM and TGW who sell sex are consistently among the highest risk subgroups for HIV infection within key populations [9-11].

Pre-exposure prophylaxis (PrEP) is a powerful prevention tool with demonstrated efficacy and effectiveness among MSM and some promise of effectiveness among TGW [12] and has been met with high levels of anticipated acceptability among both populations [13]. Yet, observational studies, demonstration projects, and program data indicate that uptake, retention, and adherence to PrEP is insufficient in many settings [14]. The subset of young MSM and TGW who sell and exchange sex with men are at exceptionally high risk for acquisition and transmission but have been studied less than other MSM and TGW, making development and assessment of HIV prevention packages particularly urgent for these populations [1]. As significant numbers of young MSM and TGW may occasionally sell sex and may not identify or be organized as sex workers, outreach, engagement and prevention tools, and approaches must be tailored for these individuals to fit their dynamic risks [9].

Thailand has a history of long-standing community partnerships with organizations that advocate on behalf of populations that sell or exchange sex. This, coupled with the regional HIV epidemiology and cultural openness to the discussion of sex work and other forms of exchanged sex, makes Thailand an important setting for implementation science research of combination HIV prevention approaches with antiretroviral (ARV) drug–based components among young MSM and TGW who exchange sex. A large observational cohort of MSM and TGW in Bangkok has demonstrated high and sustained HIV incidence (11.1/100 person-years [PY]) among 511 young (aged 18-21 years) MSM and TGW who sold or exchanged sex. Incidence was elevated despite regular HIV testing and counseling, condom and lubricant provision, care for sexually transmitted infections (STIs), and a national program of universal and free ARV access [15]. This has direct relevance to HIV epidemics underway among young and minority MSM and TGW in multiple settings. Understanding how to reduce HIV transmission rates in these contexts is a shared scientific challenge with common features at biological, behavioral, and network levels [16,17].

### Study Objectives

The Combination Prevention Effectiveness (COPE) study for young (aged 18-26 years) cisgender MSM and TGW described here uses a combination prevention approach that was developed based on a modified Social Ecological Model (SEM). The SEM posits that individual-, couple-, network-, community-, and policy-level factors all impact an individual’s risk for HIV and also likely affect uptake, adherence to, and cessation of combination HIV prevention modalities among young MSM and TGW [18]. To address the multilevel factors, the combination package of individual-level condom and lubricant distribution, HIV testing, and PrEP provision was developed and coupled with a structural community empowerment intervention. The package was made available in Thailand in 2014, when the Thai government was reviewing methods/mechanisms to provide daily PrEP for use among key populations as part of the National HIV programming for MSM and TGW [19]. The COPE study aims to assess the effectiveness and cost-effectiveness of this prevention intervention among HIV-uninfected young MSM and TGW who are engaged in sex work or who exchange sex in urban Thailand.

## Methods

### Summary

This study uses a mixed methods exploratory sequential design to develop and test the effectiveness of a combination HIV prevention intervention, with or without daily oral PrEP, in preventing HIV acquisition among young cisgender MSM and TGW who sell or exchange sex. The intervention and research methods are underpinned by ongoing community mobilization and informed by formative research, which is followed by an open-label implementation science study of intervention effectiveness and costing analysis.

The specific aims of this study are as follows: Aim 1 conducts formative research to assess the acceptability, feasibility, and optimal design of a combination HIV preventive intervention with and without daily oral Truvada PrEP for HIV-uninfected MSM and TGW in Bangkok and Pattaya, with a focus on MSM and TGW who currently sell sex to other men or who have sold sex in the previous 12 months. Aim 2 assesses the effectiveness of an open-label combination HIV preventive intervention with and without daily oral Truvada PrEP with mobile phone–based SMS support, among young MSM and TGW. Sub-aim 2.1 analyzes and describes the associations of sex work with sexual risk taking among young MSM and TGW, including numbers and types of partners and condom use, and assesses the impact of sex work, including transitions in and out of sex work, on PrEP uptake, adherence, and cessation among young MSM and TGW. Aim 3 assesses the incremental costs associated with combined HIV prevention with PrEP use, the number of infections averted through PrEP use, the discounted treatment costs saved, and the discounted disability-adjusted life years (DALYs) averted to assess whether the use of PrEP relative to the standard intervention is cost-saving, highly cost-effective, cost-effective, or not cost-effective.

Community mobilization principles guide the study design and implementation to optimize implementation, the value of results obtained, and translation of study findings to practical use. At the foundation of the community engagement is ongoing input and coordination with local community-based organizations (CBO) who work with MSM and TGW communities in Bangkok and Pattaya.

### Setting and Context

In Thailand, sex work is not legal, but there are open and clear channels for community engagement. There is an openness and accessibility to those practicing sex work, and a substantial sex tourism industry, estimated to be US $6.4 billion a year as of 2015 [20,21]. The COPE study is centrally focused on PrEP implementation and effectiveness for young MSM and TGW who are engaged in sex work or otherwise exchanging sex for money, substances, or other items of value (here forward broadly referred to as *exchange*) in Bangkok and Pattaya, and who are at highest risk for HIV acquisition. As the early period of exchanging sex is a very high-risk period for HIV acquisition, this study aims to address risk for young MSM and TGW who are newly entering sex work or exchanging sex.

The study was initially proposed for MSM engaged in the sex work/exchange but was expanded to include TGW in response to community requests. Sexual and gender identities are not distinct in Thailand as they are in Western cultures, and although we use terms MSM and TGW that are accepted in the scientific literature, these broadly include approximately 15 unique identities that encompass both sexual and gender identities and expression [20,22]. MSM and TGW who exchange sex have overlapping communities and venues and are often served by the same organizations; thus, it was determined to be appropriate and equitable to extend this intervention to both groups.

This research effort is highly integrated with Thailand national policy development around the integration of PrEP into national HIV programming for MSM and TGW. The government provides free PrEP to key populations through community-based programs but has called for the development of appropriate implementation models for PrEP and cost-effectiveness analyses to support policy decisions on government-supported PrEP distribution for key populations. As a result, several community-based clinics have been established and trained to provide PrEP, including clinics operated by the Rainbow Sky Association of Thailand (RSAT) and Service Workers In Group (SWING) Foundation.

The COPE study is supported by a joint collaboration between the Thai Ministry of Public Health (MOPH) and the United States Centers for Disease Control and Prevention (US CDC; known as the Thai-US Collaboration [TUC]) and in partnership with Mahidol University, Emory University, Johns Hopkins University School of Public Health, CBOs, SWING, RSAT, and the Asia Pacific Coalition on Male Sexual Health (APCOM). Primary implementation is at Silom Community Clinic (SCC) Clinical Research Site, operated by TUC. All recruitment and study implementation activities are done through SCC, SWING, and RSAT sites in Bangkok and SWING in Pattaya. Each study site has a site coordinator who provides oversight on study activities and communicates regularly to the multisite coordinator and study coordinator. Research teams also provide a physician-in-charge who is responsible for all clinical activities and care provided at each study site.

### Community Mobilization

Community engagement principles and mobilization activities are intended to create an enabling HIV prevention environment for young MSM and TGW. For this study, our partners SWING and RSAT serve as the initial conduit for community engagement. These partners have extensive experience in providing HIV prevention programs to gay, bisexual, transgender, and other MSM, including those who exchange sex.

The chief community mobilization activity is an ongoing series of forums targeting various venue types, age groups, and geographic subzones that are led by CBO staff in conjunction with young MSM and TGW. Point persons at participating venues who introduce the study also aid in raising awareness about and invite participants to community engagement activities. Forums can range in size from 5 to 50 community participants, depending on the venue and focus. In some cases, activities may be combined with larger community events, such as PRIDE and Songkran events, facilitating more space and engagement of more participants. Community engagement activities are ongoing through the life of the study, although participants in the community engagement activities can determine the frequency in which they participate in the events. Community mobilization facilitates scientific literacy specific to PrEP and acceptability and engagement with this HIV prevention strategy, building on gaps and concerns identified by young MSM and TGW in an earlier qualitative phase of this study. We anticipate that the community mobilization process will create an enabling, supportive environment to collectively identify and address broader issues of concern to the young MSM and TGW communities.

Through these forums and supportive activities, young MSM and TGW may (1) reflect on PrEP implementation, including any perceived implementation barriers and concerns about acceptability and uptake across the SEM, and (2) identify, prioritize, and address broader problems facing the community. We anticipate that issues will be raised across the SEM, for example, risk perception, culturally competent care, stigma related to HIV and/or PrEP, and structural risk sources such as owner/manager issues. In turn, the forums provide a mechanism for mounting a community-led response, by which we anticipate that young MSM and TGW will take on increasing leadership roles, and the group will iteratively identify problems and develop shared goals and activities deemed to be meaningful, safe, and feasible. The meetings also provide ongoing feedback to identify PrEP-related questions or concerns among forum members, including the ethical considerations noted in the “Ethical Review” section of this paper. This intervention element targets the community level; thus, activities are not limited to study participants and are open to all young MSM and TGW who meet age eligibility criteria.

### Formative Research

Formative, qualitative research among young MSM and TGW engaged in sex work informs the development of recruitment and advertising campaigns, study methods, combination prevention package, and community empowerment methods. Key informant interviews (KIIs) provide preliminary information about the acceptability, feasibility, and optimal ways to deliver combination of HIV preventive interventions to these communities.

Qualitative data collection focuses on 2 groups: (1) young MSM and TGW with a recent (within previous 12 months) history of having sold or exchanged sex and (2) the management and other staff in sex work venues. To participate in the qualitative research, MSM and TGW must meet the following eligibility criteria: aged 18 to 26 years, male assigned sex at birth, Thai nationality, able to speak and read Thai, report selling or trading sex to men in the last 12 months, currently living in the Bangkok metropolitan area or Pattaya, and willing to give written informed consent to participate.

SWING estimates that there are more than 65 venues with staff and management already participating in HIV outreach and education activities from which participants can be drawn. Two to three staff are invited to participate from each participating venue. Venue staff and management are eligible if they are aged greater than or equal to 20 years, Thai nationality, worked for one of the identified sex work venues for at least 12 months, and willing to give written informed consent.

Recruitment comprises a maximum of 132 participants (76 young MSM and TGW who have exchanged sex and 56 venue staff/managers) for KII. In this study, each participant can provide one KII only. Participant numbers and numbers of events will be determined when saturation of information has been achieved, insofar as additional interviews do not provide additional useful information beyond what has already been gained through prior interviews.

A short questionnaire is used to assess key sociodemographic characteristics and risk behaviors. A qualitative interview guide is used to guide conversations. Guides for use among young MSM and TGW include the following domains: knowledge and attitudes toward existing HIV prevention services; perceived accessibility of existing services; barriers to service use including stigma, knowledge, and local practices around issues concerning sex, and sex and substance use; perceived structural/contextual factors influencing sexual decision making; appropriate messaging/logos for educational materials; appropriate mechanisms for recruitment into the subsequent effectiveness evaluation; and feasibility and acceptability of community mobilization activities including perceived willingness to participate across subpopulations, and optimal timing, location, and scope of community mobilization activities. Guides utilized for KIIs with venue staff include the following research domains: knowledge of the local context of sex work and exchange; perceived ability and barriers to provide health care services to young MSM and TGW in venues or off-site; attitudes and perceptions of venue staff regarding HIV status of employees, of HIV testing, and of worker autonomy, privacy, and choice of HIV status disclosure, and acceptability and perceived barriers to community mobilization activities. All interviews are anonymous, conducted in Thai, and recorded using digital audio recorders, with participant consent. Participants are offered a modest reimbursement for travel and their time, 800 Thai Baht (THB) per session (approximately US $25).

Using the Thai transcripts and English-language translations, study staff conduct coding of text in Atlas.ti (Scientific Software Development). Study staff review transcripts to develop an initial coding scheme specific to the chief aims. An initial set of transcripts for each group (ie, young MSM and TGW and venue staff) are coded by 2 separate reviewers on the coding team; once agreement on core constructs is achieved, the coding team independently codes the remaining interviews, while staying in communication to identify and discuss emergent themes. For this formative study, field notes and preliminary results are also consulted as needed for timely use of these data.

### Intervention Effectiveness Study Design

**Figure 1:**
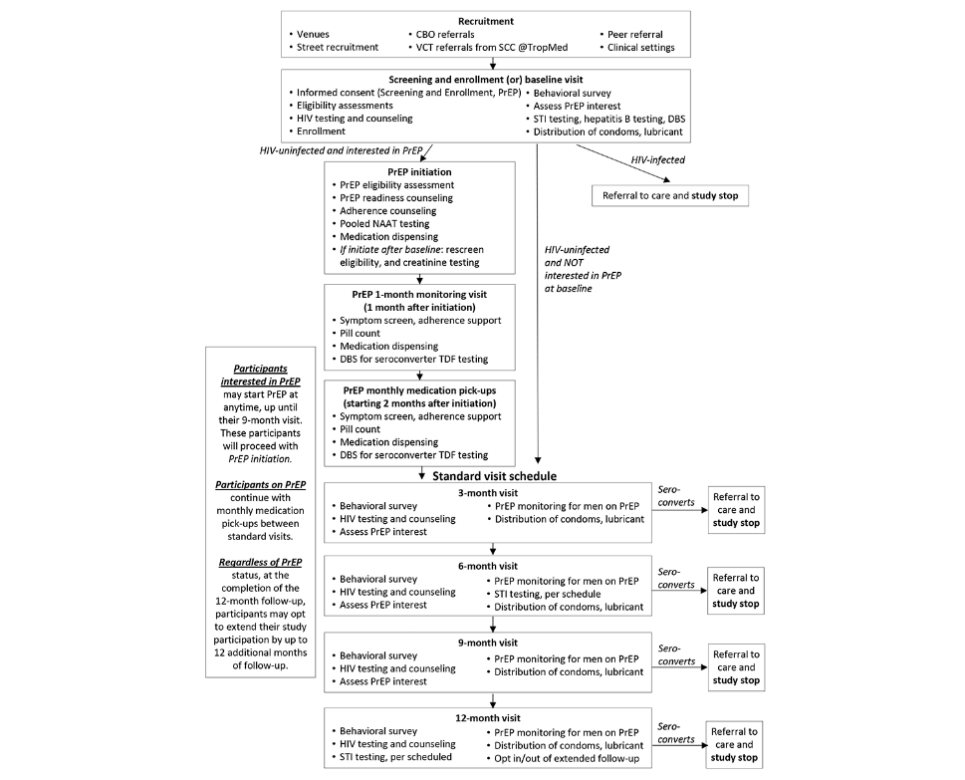
Study flow diagram and participant study visits for the Combination Prevention Effectiveness study for young men who have sex with men (MSM) and transgender women (TGW), Thailand (2015–2020). CBO: community-based organization; DBS: dried blood spots; NAAT: nucleic acid amplification test; PrEP: pre-exposure prophylaxis; SCC: Silom Community Clinic; STI: sexually transmitted infection; VCT: voluntary counseling and testing.

The study is an open label, nonrandomized assessment comparing the relative effectiveness of a combination preventive intervention with and without daily oral tenofovir/emtricitabine (FTC; Truvada) PrEP with adherence support. We follow those young MSM and TGW who choose to initiate PrEP as part of the combination package, and those who agree to participate but do not choose to start on PrEP, for whom all other intervention components will be offered. The outcome will be assessed using a person-time approach, assessing HIV incidence densities among young MSM and TGW on and off daily oral PrEP over 12 to 24 months of follow-up. Participants are enrolled for a standard follow-up period of 12 months; however, given the importance of remaining on PrEP for people in high-risk contexts, we offer the option to re-enroll for an additional 12 months. All participants, regardless of PrEP status complete quarterly study visits and weekly, brief behavioral SMS surveys. Participants who initiate PrEP are required to attend monthly medication pickup visits. Figure 1 displays the flow diagram of the study with a brief description of study visit activities.

### Combination Prevention Intervention

All participants will receive regular (every 3 months) HIV testing with risk-reduction counseling. Participants who have negative HIV rapid test results are counseled on the benefits of using PrEP in preventing HIV infection. This counseling includes a clear message on the efficacy of PrEP, discussion of barriers to initiating PrEP, and potential solutions to address these barriers. Although participants are not required to initiate PrEP as part of the study, study staff regularly promote the benefits of PrEP uptake. Condom and condom-compatible lubricant distribution occur at every study visit, including monthly medication pickups for those using PrEP. Syphilis, rectal gonorrhea, and chlamydia screenings with treatment for all identified infections are conducted every 6 months beginning at the baseline visit.

### Daily Oral Pre-Exposure Prophylaxis

Daily oral PrEP with adherence support is offered to all participants (open label). Tenofovir disoproxil fumarate (TDF) and FTC (locally known as Teno-EM) is approved for PrEP in Thailand. The PrEP drug for the COPE study is Truvada, which contains identical chemical components as Teno-EM. The tablet includes 200 mg of FTC and 300 mg of TDF. Truvada is approved for treatment in Thailand (registration number: 2C 28/2551[NC]) and is manufactured and donated by Gilead Sciences for the COPE study. Truvada is a once daily oral tablet taken with or without food.

Participants who take PrEP are counseled on the mild side effects that are expected with Truvada including nausea, headache, abdominal pain, rash, and flatulence. They are also counseled to avoid medications with potential drug interactions as outlined in the 2014 Clinical Practice Guideline, CDC [23]. These include acyclovir, valacyclovir, cidofovir, ganciclovir, valganciclovir, aminoglycosides, high-dose or multiple nonsteroidal anti-inflammatory drugs, or other drugs that reduce renal function.

Participants on PrEP have monthly medication pickups for the entire duration they are using PrEP. At each monthly medication pickup, participants return to their selected clinic for the following procedures: unused medication drop-off, tablet count adherence, dispensing of one 30-tablet bottle of Truvada; collection of dried blood spots (DBS) for storage; assessment of signs and symptoms of acute HIV infection; assessment of side effects associated with PrEP use; and adherence assessment and counseling, informed by weekly SMS survey responses.

### Short Message Service–Based Adherence Support

SMS-based adherence support is provided as part of the combination package with PrEP, in addition to standard adherence counseling. Adherence has been a significant challenge to the efficacy of PrEP and may be an important challenge for PrEP effectiveness. A 2014 systematic review and meta-analysis demonstrated that text-messaging interventions effectively supported adherence to PrEP more than control groups and were also associated with improved viral load and/or CD4 counts for individuals on antiretroviral therapy (ART) [24]. Text message reminders that were sent less frequently than daily tended to have greater effects [24]. Weekly SMS adherence prompts are therefore offered to participants choosing PrEP. SMS adherence prompts are dispatched through FrontlineSMS, with additional management by the research team. For participants agreeing to weekly prompts, participants’ FrontlineSMS profiles are configured for adherence messaging at the visit in which they initiate PrEP. The FrontlineSMS participant profile can be updated for any changes in PrEP use or for changes in timing of prompts, mobile device numbers, or carrier.

### Study Population

Eligible young MSM and TGW participants must meet the following key criteria: assigned male sex at birth; aged 18 to 26 years; HIV-negative at baseline testing; self-report that they have sold or exchanged sex to cis-gender men for money, drugs, or other goods in the past 12 months; self-report living in greater Bangkok metropolitan area or Pattaya; are Thai citizens; and are willing and able to complete study instruments. Participants do not have to self-identify as MSM or TGW, although such terms will be used in recruitment scripts to attempt to recruit participants from these populations.

Additional eligibility criteria for PrEP includes the following: HIV-negative at the visit (if acute HIV symptoms, can start PrEP at a subsequent return visit if the nucleic acid amplification test [NAAT] is negative); estimated creatinine clearance (eCrCl)≥60 mL/min; no major known allergy to tenofovir and/or FTC or the combination of the two; and able and willing to follow the PrEP prescribing guidelines.

### Recruitment and Consent

Convenience sampling methods are used, recruiting participants from a variety of venues including bars, clubs, saunas, brothels, karaoke parlors, cabarets, street-based locations, and other venues frequented by young MSM and TGW. These currently include 68 venues mapped by SWING in their 2015 census of venues in Bangkok. Outreach and recruitment are conducted by staff who have been trained in confidentiality and study procedures.

**Figure 2:**
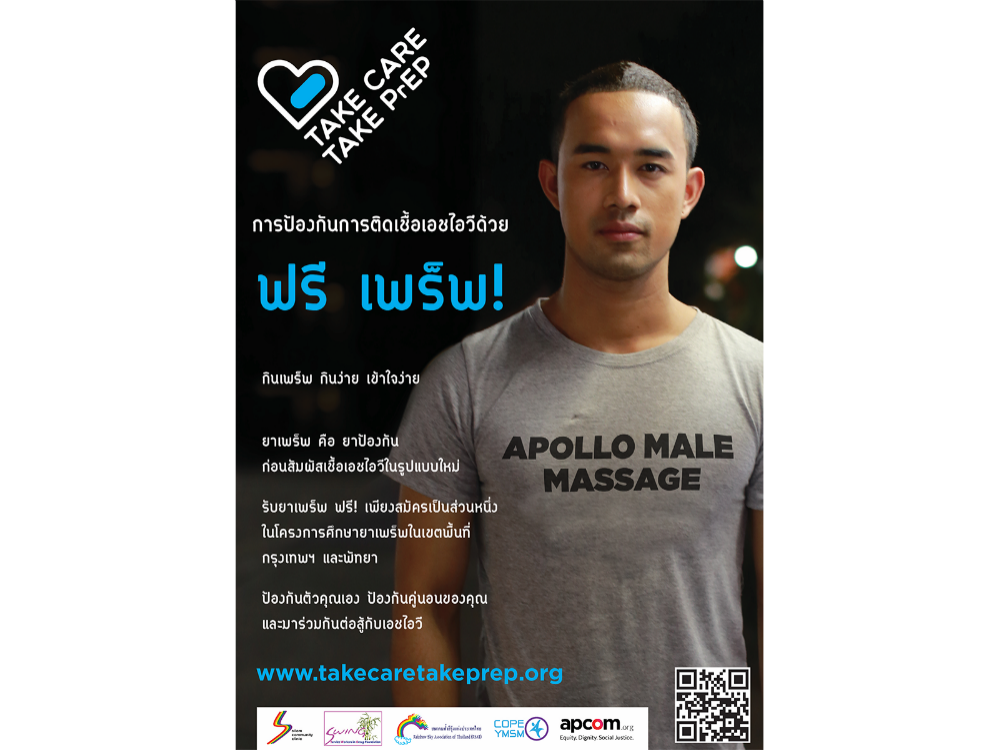
Promotional flier for the Combination Prevention Effectiveness Study for young men who have sex with men and transgender women, Thailand.

Recruitment also occurs online, using materials such as videos and fliers developed in collaboration with the community and produced by APCOM [25]. Staff are trained on how to avoid coercing individuals to participate in the study. Recruitment materials are posted on dating apps, Facebook, and other frequently used online venues. These recruitment materials also serve to raise awareness about and increase demand for PrEP. In addition, the study website, study clinic websites, and Facebook pages are used to provide community-wide access to information about services and the goals of the study [26]. All interested individuals are invited to contact the study staff for preliminary screening and to learn more about the study. Figure 2 displays an example study flier for MSM and TGW.

Study participants are invited to refer peers to the study. Participants are provided study information cards that they can distribute to potentially eligible peers who are MSM or TGW who are newly engaged in sex work or who have recently exchanged sex. Referred peers may be enrolled into the study upon meeting inclusion criteria. Participants who successfully recruit peers are compensated per similar standard practices implemented in the LINKAGES program in Thailand to support peer referral to HIV testing and care [27]. Study participants will be reminded to maintain confidentiality before referring peers and distributing information cards.

Candidate participants are not screened in person during recruitment events or peer or community referrals. Interested individuals are provided study information cards with a phone number, email address, and link to a website with more information about the study eligibility criteria, purpose, available clinics, and clinic hours. Interested individuals may indicate whether they would prefer to call the study offices to schedule an appointment at their own convenience or to provide their phone number and be contacted by study staff. During the initial phone call with study staff, candidate participants are informed of the study purpose and eligibility criteria and determine whether or not they would like to proceed with study recruitment. Aggregate data are collected from these phone calls on the number of individuals contacted or who contacted the study, those that were scheduled, and reasons not scheduled.

Consent is obtained from recruited young MSM and TGW, and they are screened for eligibility in private at one of the study sites. Those who are eligible are enrolled in the study. The screening visit includes an assessment of demographic criteria and rapid HIV testing. Participants identified with HIV infection complete their screening visit, including facilitated referral to care but do not attend any study follow-up visits. Participants who are determined to be living with HIV at their screening visit do not count toward the target sample size. At baseline, participants are also screened for hepatitis B and may initiate the vaccine series during follow-up, if susceptible.

Participants who are identified as being HIV-uninfected by rapid HIV testing are prospectively enrolled into the study. Enrolled participants are offered a combination prevention intervention package, with the option to initiate daily PrEP. Prospectively enrolled participants have an initial follow-up period of 12 months; participants who complete their 12-month follow-up period, regardless of PrEP initiation status, are offered the option of further extending their follow-up for another 12 months if the study timeline permits.

### Study Visits

Participants, regardless of PrEP initiation, have standard follow-up study visits every 3 months (quarterly) that include HIV testing and counseling, condom and lubricant distribution, and surveys using computer-assisted self-interview (CASI) or with audio-assisted CASI (ACASI) technology. Participants are screened for syphilis, rectal gonorrhea, and chlamydia at their baseline study visit and every 6 months (semiannually) thereafter. Table 1 describes data collection activities that take place quarterly or semiannually, regardless of PrEP use. Through these visits and biological and survey outcomes, the study staff are able to assess the acceptability, uptake, adherence, and effectiveness of the combination intervention package for young MSM and TGW.

At any time during the study period from baseline to the 9-month visit, participants can be screened for PrEP eligibility with point of care creatinine testing. If eligible, they can initiate PrEP at that visit. Ineligible participants, or participants who do not express an interest in initiating PrEP during the initial 9-month study period may continue study participation as non-PrEP participants. Non-PrEP participants complete quarterly study visits and receive all standard procedures, including the CASI, HIV testing, hepatitis and STI testing, and DBS collection.

All participants initiating PrEP complete a PrEP monitoring visit 1 month after PrEP initiation to assess for acute HIV symptoms and provide adherence support. Participants on PrEP are then asked to complete monthly visits for medication pickup. Medication pickups can be combined with quarterly study visits during months in which they are due.

Participants can initiate, discontinue, and restart PrEP at any point during the study period. Changes to PrEP use can be communicated during any study visit, via weekly SMS surveys, or can be communicated to study staff outside of study visits. Reasons for discontinuing PrEP are recorded. Participants who restart PrEP after more than 2 weeks cessation must be rescreened. Study investigators consider someone stopped on PrEP if the participant’s weekly SMS adherence measures indicate that he/she had 2 consecutive weeks with 0 to 3 doses taken, or if the participant has >16 pills remaining from the month during a medication pickup. PrEP rescreening includes HIV testing and an assessment of willingness to commit to PrEP adherence. Participants who stop PrEP may continue in the study and continue with their remaining quarterly study visits. Participants who permanently stop PrEP or who do not want to immediately restart PrEP are instructed to return all Truvada as soon as possible.

Participants who seroconvert during the study period complete a close-out survey, receive facilitated referral to care with assistance by the case manager or site coordinator at each site, are discontinued from the study and do not attend subsequent study visits.

**Table 1:** Description of quarterly and semiannual study visits for the Combination Prevention Effectiveness Study for cisgender men who have sex with men and transgender women, Thailand.

Study visit	Description
Quarterly study visits (every 3 months)	Confirm participant contact and locator information Administer CASI^a^ behavioral questionnaire Assess participant experience and impacts of study participation (including adverse events and social harms) Screen for signs and symptoms of acute HIV infection Provide HIV testing (including pooled NAAT^b^ testing if the participant has symptoms of acute HIV infection) Collect DBS^c^ for storage Distribute condom and lubricant
Semiannual study visits (every 6 months)	All procedures conducted at quarterly study visits Syphilis testing Rectal swab collection for rectal gonorrhea and chlamydia testing Serum creatinine measurement and estimated creatinine clearance calculation (for participants on PrEP^d^)

^a^CASI: computer-assisted self-interview.

^b^NAAT: nucleic acid amplification test.

^c^DBS: dried blood spots.

^d^PrEP: pre-exposure prophylaxis.

### Behavioral Survey

Sociodemographic and behavioral data are collected from all participants at baseline and at each follow-up visit using a standard survey. The survey is administered using CASI or ACASI depending on participant preference. Use of CASI on a standard personal computer or tablet occurs in a structurally secluded and quiet area at the study clinic to ensure confidentiality and to encourage candid disclosure of risk behavior. Trained staff are available to answer questions pertaining to the survey questionnaire.

Survey questionnaires span the following domains and are administered over the course of the study: sociodemographic; knowledge, awareness, and use history of PrEP, postexposure prophylaxis, or ART; health care utilization; engagement in the lesbian, gay, bisexual, and transgender (LGBT) communities; postrecruitment questions; sexual risk behavior; stigma; substance abuse; and mental health. Additional information on the focus of each domain is provided in Table 2.

**Table 2:** Sociobehavioral survey domains, descriptions, and measures for the Combination Prevention Effectiveness study for young cisgender men who have sex with men and transgender women, Thailand.

Domain	Description
Sociodemographic	Information collected in this domain includes, but is not limited to, age, education, income, mobility, and insurance status.
PEP^a^/PrEP^b^	Information collected in this domain focuses on initial knowledge, awareness, acceptability of and attitudes about PEP/PrEP, and history of use (including recreational use and source of PEP/PrEP).
Sexual risk behavior	Information collected within this domain includes information on number and types of partners (sex work client, nonpaying casual, and stable relationship), frequency and types of sexual acts, condom use during intercourse, and location of the sexual experiences. This includes details on sex work (initiating and stopping), average number of clients per night and week, average number of nights worked per week, and regular/noncommercial sexual partners.
Health care utilization	Information collected in this domain includes information on health care utilization and services to assess engagement at baseline. Information in this domain is also collected at the final visit to assess any supplemental services that have been accessed outside the study.
Engagement in the MSM^c^, TGW^d^, and sex work communities	Information collected in this domain includes information on knowledge of and engagement within community forums, PrEP-related contact with outreach workers, social support, connection and collective efficacy within the LGBT^e^ community and in the sex worker community, and perceived support for PrEP and related topics within these communities. Receipt of condoms and contact with outreach workers will also be assessed. This domain allows the team to evaluate the effectiveness of the community mobilization intervention component.
Stigma	Information collected in this domain is focused on self-stigma (internalized homonegativity). This survey domain is adapted from Herek et al [28].
Substance abuse	Information collected in this section includes information on any substances used (amphetamine type substances, alcohol, etc) particularly around sexual experiences and frequency of substance use.
Mental health	The information collected in this section focuses on brief depression, posttraumatic stress disorder, and anxiety screeners using the Substance Abuse/Mental Illness Symptoms Screener [29,30].

^a^PEP: postexposure prophylaxis.

^b^PrEP: pre-exposure prophylaxis.

^c^MSM: men who have sex with men.

^d^TGW: transgender women.

^e^LGBT: lesbian, gay, bisexual, and transgender.

### Weekly Short Message Service Surveys

An open-source software, FrontlineSMS, is used to survey participants via text messages (SMS). FrontlineSMS is able to send and receive messages, respond to messages, and trigger other events. FrontlineSMS is programmed with the cell phone number of the study participant after study enrollment, informed consent, and selected preventive option. Through FrontlineSMS, weekly, brief behavioral surveys are sent to participants to obtain information on exchange sex and condom use in the last week. For participants currently using PrEP, additional measures are used to collect information on when they last took PrEP and the number of doses of PrEP taken within the last 7 days. For those reporting less than 4 doses, additional questions inquire about intention to continue taking PrEP, and, if applicable, reason for discontinuing PrEP use. To ensure participant privacy, SMS surveys are only unlocked by a unique code assigned to each participant upon registration. Participants who are unable to immediately complete an SMS survey because of privacy concerns may opt to postpone their response within an allowable timeframe. Participants will receive cash compensation (30 THB, equivalent to approximately US $1) for responding to reminders and to reimburse cellular data costs.

### Monthly Medication Pickups

Additional study data are collected during monthly medication pickups. These include count of unused tablets and DBS collection for storage and testing for tenofovir diphosphate if the participant becomes HIV-infected. Participants are requested to bring PrEP bottles with them to their monthly medication pickup, which also permits appropriate destruction of unused medication. Participants can receive new PrEP refills if they do not have their unused tablets with them, and the study team submits a memo-to-file. We collect both tablet-count adherence data (measured as number of unused tablets at the end of the month) and self-reported SMS adherence for the purposes of having the most complete and comprehensive measures of adherence.

### Biologic Measures

One standard blood draw is done for all participants, as it is done before knowing HIV status. Blood collected is used for rapid HIV testing as outlined below. If HIV rapid test(s) is positive, blood is then tested for CD4 count and syphilis only. If rapid tests are negative, the following tests are conducted: syphilis, hepatitis B, and DBS for storage. Table 3 summarizes all laboratory tests conducted for COPE participants at each study visit.

DBS samples are used to measure study drug levels for those initiating PrEP and subsequently become HIV infected. If participant initiates PrEP at the visit or displays symptoms of acute HIV infection, a pooled NAAT test is also conducted. Study staff store blood specimens collected from quarterly study visits at least through the end of the study. Additional DBS collected from participants on PrEP at each study visit and medication pickup after PrEP initiation are stored in a −20°C freezer after drying. DBS specimens for participants prescribed PrEP who seroconvert will be shipped in bulk in dry ice to the University of Cape Town laboratory quarterly for testing. DBS from these participants will be assayed to measure levels of intracellular tenofovir diphosphate.

**Table 3:** Biologic measures taken per participant, by Combination Prevention Effectiveness Study visit.

Study visit	Biologic measure
Baseline visit	HIV rapid testing Pooled NAAT^a^ Syphilis Hepatitis B DBS^b^ CD4 (if HIV-infected) Creatinine
Follow-up visit with STI^c^ screening only	HIV rapid testing Pooled NAAT Syphilis DBS CD4 (if HIV-infected)
Follow-up visit with STI and creatinine screening (for participants initiating PrEP^d^ outside of baseline only)	HIV rapid testing Pooled NAAT Syphilis DBS CD4 (if HIV-infected) Creatinine
Monthly PrEP pickup (for PrEP participants only)	DBS

^a^NAAT: nucleic acid amplification test.

^b^DBS: dried blood spots.

^c^STI: sexually transmitted infection.

^d^PrEP pre-exposure prophylaxis.

### HIV Testing

HIV testing is completed at each regular study visit at 3-month intervals. For participants initiating/restarting PrEP or for those who display signs or symptoms of acute HIV infection at a study visit, HIV testing consists of a rapid antibody test supplemented by reflex pooled NAAT testing for antibody-negative specimens. Those with symptoms of acute HIV infection are restricted from starting on PrEP until their NAAT test results return negative. Those without symptoms of acute HIV infection can initiate PrEP if interested and eligible; if the NAAT result is positive these participants are contacted, instructed to stop taking PrEP, and asked to return to the clinic as soon as possible to reduce usage of PrEP during acute infection. All HIV-positive results will be confirmed by additional rapid and/or NAATs, in accordance with the recommended HIV testing algorithm for Thailand.

To reduce the risk of drug dosing post-HIV infection, participants on PrEP are advised to return for HIV testing and clinical evaluation, even if outside of scheduled visits, if they experience possible symptoms of acute HIV infection including fever, myalgia, fatigue, oral ulcer, skin rash, sore throat, or headache. All participants who become HIV infected while on PrEP are offered TDF and FTC resistance (K65R, M184V) testing and are referred to HIV care and treatment. Participants not on PrEP are advised to return for HIV testing if they display any of the acute HIV symptoms mentioned above.

All participants found to be HIV-infected either during the screening process or during the course of the study are able to access HIV treatment and care services through the national health scheme in Thailand, which guarantees universal access to ARVs for Thai citizens. The case manager or site coordinator at each site will complete a facilitated referral for all participants who test positive for HIV, which includes CD4 count, referral letter, and case management as needed according to standard, site-specific practices.

### Sexually Transmitted Infection Testing

Participants are screened for syphilis and rectal gonorrhea and chlamydia at their baseline study visit and every 6 months thereafter. Rectal gonorrhea and chlamydia are assessed using either provider-collected or a self-administered rectal swab depending on participant preference. Self-administered swabs have been used in prior studies with good acceptability [31]. Participants with positive results are provided treatment either at the clinic per MOPH guidelines or are supported to get STI treatment at a local health care facility. Participants reporting signs or symptoms of other STIs are evaluated and either treated on site or referred to care.

### Hepatitis B Screening

Given the current use of Truvada in current treatment regimens for hepatitis B, all prospective participants are screened for hepatitis B infection at their baseline visit. If susceptible, they can initiate the hepatitis B vaccination series at their next visit or medication pickup. Participants initiating the hepatitis B vaccine series are referred to SCC @TropMed, or a local hospital.

### Assessment of Renal Function

A serum creatinine test is completed for all participants before PrEP initiation. eCrCl is calculated using the Cockcroft-Gault formula below.

eCrCl=[[(140 age in years)actual body weight in kg](serum creatinine in mg/dL72)]

Any participant with an eCrCl of <60 mL/min is not eligible for PrEP. PrEP users complete creatinine testing every 6 months while on PrEP.

### Cost Analysis Data Collection

Broad intervention categories include community outreach and recruitment, HIV testing, condom and condom-compatible lubricant promotion, STI testing, and PrEP distribution. Community engagement and technology are also provided to support and encourage PrEP use. Costs associated with PrEP and technology use capture a majority of the costs associated with PrEP use and are a primary component of the incremental cost-effectiveness analysis.

Study participation primarily consists of visits with procedures related to the intervention, related to research questions, or a combination of the two. However, all research-related costs are excluded in cost-effectiveness analysis. In addition, participant involvement may vary across procedures, as some may be conditional (eg, given positive test result) and some may be repeated to varying degrees (eg, adherence assessment). For each program procedure, we establish the types of resources used, the value of each unit of resource, the quantity of resources used each time the procedure occurs, including the *best estimate* of the mean and the distribution or range, and the frequency with which the procedure occurs.

The majority of procedure-based costs are expected to be variable costs, as they are consumed each time the procedure occurs. These are complemented by procedure-specific fixed costs, such as the costs associated with the development of an SMS system. In addition to procedure-specific costs, there are general costs. Fixed general costs may include facility-related costs (eg, rent and utilities), electronic equipment, software, maintenance, administrative staff, and administrative overhead. General variable costs may include personnel time not directed to specific participant procedures, including staff meetings, trainings, and supervision.

A microcosting approach [32-34] is used to directly enumerate the cost of every input used in the intervention, such as staff time spent on each intervention-related activity, facility space, equipment, materials [32,34-36], and all in-kind contributions.

Procedure-specific resources are identified from walk-throughs of procedures, surveys and interviews with program staff, and review of program records. A walk-through consists of a member of the costing team performing a real-time observation of study procedures during a participant visit, beginning with initial referrals of potential participants to the study. Visits (and procedures) include the following: recruitment; eligibility screening; enrollment; PrEP education, offer, and eligibility screening; PrEP initiation; PrEP monitoring; monthly PrEP medication pickup; and quarterly study visits. Walk-throughs of visits or procedures also evaluate contingencies that may depend on test results, eligibility, or participant preferences. Through this process, the costing team identifies activities conducted and materials consumed through each procedure. Walk-throughs from the perspective of program staff are also conducted to help identify ancillary activities such as procedure-related administrative tasks. Resources needed for the prospective component of the study are assessed through observation of practices and not through human subjects activities.

Fixed and variable general costs are identified through surveys completed by program managers. Interviews with program staff and management are used to confirm the use of identified resources and to identify any additional resources that may have been missed through this process. Budgetary and other administrative records are also reviewed to identify any additional resources.

The number of units consumed during procedures is established through a combination of the abovementioned walk-throughs of procedures, direct observation of procedures, periodic surveys with study staff, and routine data collection. Data on the number of units consumed per procedure are complemented by data collection on the number of times different iterations of a procedure occurs, the number of participants receiving procedures, and the PY on PrEP per participant. Collection of these data is integrated into data collection for program evaluation (ie, study records) and data collection for the provision of clinical services (ie, participant clinical records).

From the costing data, we estimate for each procedure the mean procedure-specific cost per occurrence of the procedure. The frequency of procedures informs our estimations of the mean general costs per participant, the total costs across all participants, and the incremental cost per person-year on PrEP. The granularity of the costing data also allows estimates of alternative protocols, such as PrEP with and without adherence support, and PrEP with a reduced biological testing protocol. Data collection instruments and methods were pilot-tested and refined during the formative phase and reviewed and validated by the study partners.

### Quality Assurance/Quality Control Measures

Study coordinators regularly inspect study facilities and documentation (eg, informed consent forms, clinic and laboratory records, other source documents, and case report forms), as well as observe the performance of study procedures. Investigators also allow inspection of all study-related documentation by authorized representatives. A site visit log is maintained at the study site to document all authorized inspection visits and audits.

On-site study monitoring is performed by site coordinators and the multisite coordinator. Monitoring includes verification of compliance with the consenting process and other human subjects guidelines; assessment of adherence to the study protocol, study-specific procedures manual, and local counseling practices; and confirmation of the quality and accuracy of information collected at the study site and entered into the study database.

All data forms are reviewed for accuracy and completeness by study staff before sending data to TUC, where data cleaning is performed. At TUC, errors identified during the data cleaning process are compiled into a quality control (QC) report and sent back to site study staff for corrections. QC reports are compiled and tracked quarterly.

### Study Retention

As this study measures person-time on and off PrEP, retention for the full study period is not a primary goal. However, to encourage participants to access prevention services and facilitate PrEP adherence, study staff make every reasonable effort to follow participants for the entire study period. A study-specific appointment and tracking system is used to ensure completion of assessments and to facilitate management of participant tracking. Tracking methods include SMS text messages, voice calls, emails, and visits to participants’ place of employment. Confidentiality for participants is maintained through outreach efforts including text messaging, phone calls, and emails that do not disclose any details of study participation and that are sent by outreach staff trained on maintaining confidentiality of participants and potential participants, using outreach method participants have selected as their preferred method (ie, SMS, voice, emails, or workplace visits).

Study staff have developed and implemented standard operating procedures for retention to minimize attrition. These procedures include thorough explanation of the study visit schedule and procedural requirements during the informed consent process and re-emphasis at each study visit; collection of detailed contact information at the study enrollment visit, and active review and updating of this information at each subsequent visit; collection of locator information from each participant, so that the participant can be found if the phone number or address changes; creation of a retention plan to maximize the level of participation in study-required visits; use of appropriate and timely visit reminder mechanisms; and immediate and multifaceted follow-up on missed visits.

### Compensation

Participants are reimbursed for their time and effort in this study and for their travel to study sites. Participants receive 1000 THB (US $35) per visit attended at 0 (baseline), 3, 6, 9, 12 months, and a courtesy award of 500 THB (US $17.50) for completing the final (12-month) visit. If the participant travels to the study site and is deemed ineligible during the screening process, he or she receives 1000 THB (US $35).

Participants receive cash compensation for responding to weekly SMS surveys. Each person receives 30 THB (US $0.83) for each SMS survey the participant completes. If a participant achieves a perfect response rate of 4 surveys per month, he or she receives a courtesy award of 100 THB (US $3.50) at the end of the fourth survey of the month.

In addition to the above compensation, participants who initiate PrEP receive 500 THB (US $17.50) at each monthly medication pickup.

To identify participants with potential acute HIV infection while being prescribed PrEP, participants with symptoms consistent with acute HIV infection are compensated 500 THB (US $17.50) if presenting to a study site before their next scheduled visit.

For participants who initiate PrEP, the total possible compensation for 1 year of follow-up is 14,360 THB (US $479), given an increased number of study visits for pill pickups. For those who choose not to initiate PrEP, the total possible compensation for one year is 8360 THB (US $279).

### Statistical Considerations

Statistical analysis uses a person-time approach to compare HIV incidence during person-time on PrEP to HIV incidence during person-time not on PrEP.

### Sample Size Calculation

This study focuses on the primary endpoint of incident HIV infection during follow-up, comparing person-time on PrEP and off PrEP. Participant uptake of PrEP, level of PrEP adherence, and cessation of PrEP use serve as secondary endpoints in this study.

As prospectively measured in the Bangkok MSM Cohort Study cohort [37], we expect that the participants will be a mixture of very high-risk MSM and TGW 18 to 24 years of age with an HIV incidence rate of 11 per 100 PY and high-risk MSM 22 to 26 years of age with an incidence rate of 5 per 100 PY. Assuming a 50/50 mixture, we expect an incident rate of 8.5 per 100 PY (range=7-10). On the basis of recent iPrEx open-label extension (iPrEx OLE) study, we assume a 50% reduction in incidence for the PrEP group versus off-PrEP group [38]. On the basis of empirical evidence, we expect that some 50% of the participants will choose to take PrEP.

The sample size of this study is 1240 participants based on the following assumptions: incidence without PrEP is 8.5 per 100 PY, a PrEP efficacy of 50%, time-on-PrEP of 50%, attrition of approximately 2% per month, power=0.8, α (one-sided)=.025, and inflation of the standard error of the hazard ratio by 0% to 20% after adjusting for confounding. From this assumption, we expect that a combined sample of 1240 (620 on PrEP and 620 not on PrEP) will be sufficient.

### Data Analysis

Each week of study participation at risk for HIV for each participant falls into 1 of 4 mutually exclusive categories of person-time: (1) on PrEP, good adherence; (2) on PrEP, poor adherence; (3) off PrEP, recent PrEP discontinuation (in last 30 days); and (4) off PrEP, no recent PrEP exposure. Individuals are considered at risk for HIV until the first positive HIV test result or until the last HIV negative test result in the case of administrative censoring, loss-to-follow-up, or another censoring event.

The primary outcome analysis will compare HIV incidence during person-time on PrEP (categories 1 or 2) to HIV incidence during person-time not on PrEP (categories 3 or 4), with a simplified analysis following the analytic methods used by other PrEP open-label studies, and a more complex analysis using marginal structural models to account for time-varying confounding. Secondary outcome analyses will explore whether HIV incidence differs between participants on PrEP with good versus poor adherence (category 1 vs category 2) and whether HIV incidence differs between participants not on PrEP who have recently discontinued PrEP use versus who do not have recent PrEP exposure (category 3 vs category 4).

A more complex primary outcome analysis will be conducted using marginal structural models, following the methods proposed by Robins et al [39] As participants self-select into the PrEP group, the difference in incidence between those on PrEP and not on PrEP may be biased due to confounding. Propensity score methods will be used for bias reduction for the estimation of the causal effect of being on PrEP on HIV incidence, an approach that is well documented in nonrandomized pharmacoepidemiologic studies [40]. This will be an as-treated analysis that will account for discontinuation of PrEP, and as PrEP initiation and cessation may occur over the course of follow-up, and participants may cease and restart sex work, potential confounders may be time-varying, further complicating the estimation of causal effects.

In marginal structural models, inverse probability of treatment and censoring weights (IPTCWs) will be used in weighted, pooled logistic regression models of HIV infection. The data for each participant are structured such that each record represents 1 person-week of time either on PrEP or not on PrEP [39]. The odds ratio from the weighted, pooled regression provides an estimate of the hazard ratio for HIV infection associated with PrEP initiation similar to Cox time-dependent regression [41,42]. The IPTCWs at time *t_i_* incorporate the estimated probability of the observed treatment history and the estimated probability of prior censoring. Creating the IPTCWs requires estimating inverse probability of treatment weights (IPTWs), inverse probability of censoring weights (IPCWs), and stabilization weights.

To estimate the IPTWs, a series of logistic regression models will be used to estimate the propensity of transition from one treatment state at time *t_i-1_* to a new treatment state at time *t_i_*. These analyses will be limited to participants who were HIV-negative at time *t_i_*, and the analyses will include time-invariant (ie, baseline) covariates, and time-varying covariates from time *t_i_* or earlier. Potential confounders to be included in the propensity score models of treatment status will be identified based on existing knowledge and based on exploratory analyses. Factors associated with PrEP acceptability among Thai young MSM and TGW include STI history, frequency of anal intercourse, HIV testing history, PrEP awareness, and insurance status [13], and factors associated with PrEP acceptability in other populations include perceived risk for HIV, use of condoms, sexual identity, education, concerns about PrEP use, and sex with casual or exchange partners [43]. Based on the estimated coefficients, the predicted probability of the observed treatment will be estimated, and this estimate will be inverted to give the inverse probability of the observed treatment at time *t_i_*, given the observed treatment at time *t_i-1_*. Prior treatment history will be incorporated by multiplying the predicted probability of treatment by the probabilities of not receiving the observed treatment at each of the previous time points.

Stabilization weights will be similarly estimated, but, in contrast, they will be based on logistic regression models including only time-invariant covariates. The IPTWs will be multiplied by the stabilization weights to improve their performance. The IPCWs will be similarly estimated and stabilized, but using transition from observed to unobserved in place of transition between PrEP use and nonuse, and the covariates used for the censoring model may differ from the covariates used in the treatment model. Finally, the product of the stabilized IPTWs and the stabilized IPCWs will provide the IPTCWs that will be used in the pooled logistic regression model to estimate the hazard ratio of HIV infection during time on PrEP and time off PrEP. The pooled logistic regression will include all time-invariant covariates used to create the stabilization weights and additional time-invariant covariates may be identified through exploratory data analysis.

The estimated log-odds of HIV infection and the standard error of the estimate from the pooled logistic will be used to evaluate whether the hazard of HIV differs between time on PrEP and time off PrEP, with the null and alternative hypotheses evaluated using the Wald statistic.

H10: The hazard of HIV during time on PrEP will not differ from the hazard of HIV for time not on PrEP: HIV hazard ratio=1.

H1a: The hazard of HIV during time on PrEP will differ from the hazard of HIV for time not on PrEP: HIV hazard ratio1.

The simplified primary outcome analysis will be conducted using Poisson regression, following the methods used by Grant et al [38]. The simplified analysis will have the benefit of easier interpretation but lack the ability to control for time-varying confounders that may be both causes and consequences of PrEP use (eg, sexual risk behavior and risk compensation).

The Poisson regression model will control for baseline covariates and include a time-varying indicator for PrEP status. The outcome will be assessed using a person-time approach, assessing HIV incidence densities among young MSM and TGW on and off daily oral PrEP over follow-up. In the Poisson regression, the Wald statistic will be used to evaluate the coefficient for PrEP status to distinguish between the null and alternate hypotheses.

H10: The HIV incident rate during time on PrEP will not differ from the incident rate for time not on PrEP: HIV incident rate ratio=1.

H1a: The HIV incident rate during time on PrEP will differ from the incident rate for time not on PrEP: HIV incident rate ratio 1.

### Cost-Effectiveness Analysis

We will estimate the incremental cost of the intervention per DALY averted, with DALYs reflecting the present value of future years of healthy life lost to morbidity/disability and future years of life lost to premature mortality. The incremental cost will be the net total cost of the intervention, excluding the cost of standard-of-care comparison condition.

This study has a multifaceted intervention design, and the emphasis of the cost-effectiveness analysis will be on the incremental cost-effectiveness of PrEP use relative to the intervention components that are not tied to PrEP use. The cost-effectiveness analysis will be in accordance with the recommendations set forth in the US Public Health Service Panel on Cost-Effectiveness in Health and Medicine [32]. We will estimate the additional cost of PrEP use versus PrEP nonuse, number of infections averted through PrEP use, discounted treatment costs saved through PrEP use, and the discounted DALYs averted through PrEP use. On the basis of these estimates, we will determine whether the use of PrEP relative to the standard intervention is cost-saving, cost-effective, or not cost-effective using societal and provider perspectives [32,35,36].

Through the primary outcomes analyses, the number of infections averted will be estimated as the difference between the number of infections that would have been expected without PrEP treatment and the observed number of infections under PrEP treatment.

Treatment costs saved per infection averted will be based on national or local estimates of costs per year for ART in Thailand, including costs for first- and second-line ARVs, laboratory tests, facilities, staff time, and opportunistic infections [44]. Total lifetime treatment costs per HIV infection will be based on remaining life expectancy and disease progression and discounted at 3% per annum [32,45].

DALYs, calculated following the methods of Fox-Rushby and Hanson [46] and Larson [47], will capture the years of life lost and disability associated with HIV infection and will be based on both the difference in life expectancy with and without HIV infection and the difference in the quality of life during years of life not lost. Future DALYs averted will be discounted at 3% per year annum.

The incremental cost-effectiveness ratio (ICER) will be based on the estimation of incremental cost (difference between the additional intervention cost and the additional treatment cost averted) divided by the DALYs averted. The intervention will be considered cost-saving if the additional intervention cost is less than or equal to the additional treatment cost averted, considered highly cost-effective if the ICER is less than gross domestic product (GDP) per capita in Thailand, cost-effective if the ICER is between 1 and 3 times GDP per capita, and not cost-effective if ICER is more than 3 times GDP per capita [48].

For the various cost-effectiveness ratio components, uncertainty intervals will be established based on primary data collection, literature reviews, and expert opinion. One-way and multivariate sensitivity analyses will be used to examine the robustness of the cost-effective estimates. In these analyses, parameters will be varied within the uncertainly intervals, and the resulting estimates will be reported. These components are expected to include estimates of resources consumed, resource costs, discount rates, treatment costs, DALYs per HIV infection averted, and infections averted. In addition, some research procedures may conceivably contribute to an intervention effect, such as monthly PrEP pill counts and weekly SMS adherence surveys. The impact of these procedures on cost estimates and cost-effectiveness will also be evaluated in sensitivity analyses.

### Ethical Review

The protocol, site-specific informed consent form, participant education and recruitment materials, and other requested documents—and any subsequent modifications—have been reviewed and approved by the following institutional review boards (IRBs) and ethical committees (ECs): Thailand Ministry of Public Health Ethical Review Committee for Research in Human Subjects; The US CDC IRB; and The Johns Hopkins Bloomberg School of Public Health IRB. The Emory University IRB defers to the Johns Hopkins University IRB, which has been documented by letter to the principal investigator (PI). The study protocol is also reviewed and approved by the Prevention Sciences Review Committee of the Division of AIDS, National Institute of Allergy and Infectious Diseases (NIAID)/National Institutes of Health (NIH; Multimedia Appendix 1).

### Safety Monitoring and Adverse Event Reporting

Study staff have established a data and safety monitoring plan. The staff ensure that interview protocols are followed, review all adverse event reports (if any) and ensure that confidentiality procedures are implemented. The larger project team meets regularly to discuss progress toward data collection goals. At these meetings, study staff prepare weekly reports on data collection and any emerging issues or potential problems regarding the Web-based survey data collection efforts.

The site PIs report in writing all serious adverse events associated with the study procedures and/or any incidents or problems involving the conduct of the study staff or subject participation, including problems with the consent processes, per IRB protocols. Drug-related adverse events described by study participants or identified during laboratory testing after initiation of Truvada (eg, nonbaseline abnormal creatinine levels) are collected and submitted to the Thailand MOPH EC, CDC IRB, and JHU IRB in accordance with individual IRB and EC requirements, and to the Thai Food and Drug Administration as per their requirements. Adverse events severity is graded following sponsor guidelines [49]. Study participants are provided instructions for contacting the study site staff to report any negative medical occurrences they may experience. Where feasible and medically appropriate, participants are encouraged to seek evaluation where a study clinician is based; in the event this is not possible, and participants receive care elsewhere, they are asked to follow-up at SCC @TropMed for further evaluation and determination whether adverse-event reporting is necessary.

In the context of this effectiveness study of an approved drug (tenofovir and FTC) in Thailand, adverse events, should they occur, are likely to be the social harms associated with disclosure of status as an MSM or TGW, a sex worker, as having HIV infection or at risk for HIV infection, or as being on PrEP. The study team regularly assesses each of these social harms through the surveys, at monthly PrEP medicine pickups, and through regular meetings with the community advisory board. Study staff are trained on social harm counseling and monitoring before study initiation and undertake additional efforts to minimize social harms to participants. Study staff record information pertaining to the event on the Social Harm Case Report Form and provide counseling with proposed solutions or refer participants to personnel or organizations with this specific expertise. Study staff make every effort to provide appropriate care and counseling to the participant and offer referral to appropriate resources, as needed, for the participant’s safety. Study staff follow up about the social harm event at every study visit and record updated information onto the Social Harm Case Report Form. Social harms that are determined by the study investigators to be serious or unanticipated are reported to the responsible IRBs, or according to their individual IRB requirements.

## Results

### Summary

As of January 2019, formative research has been conducted, and the intervention effectiveness study has launched in Bangkok. Community engagement and empowerment efforts also launched during the formative phase and are ongoing throughout the study.

### Formative Research

Formative research has been conducted in Bangkok and is ongoing in Pattaya. A total of 41 young MSM, 21 young TGW, and 24 venue staff and managers have completed KIIs (Table 4). These interviews provided insight into the context of sex work and exchange sex in Bangkok and Pattaya, perceptions and understandings of PrEP use in these contexts, as well as informed recruitment methods and study implementation. Although qualitative research and analysis has been ongoing, debriefing sessions and notes have been used to inform study methods. Specifically, formative findings were considered in the development of the survey (length, mode of administration, terminology, and measures), study branding and marketing (logos, images, content, and media platforms), and recruitment/retention methods across subgroups and venue types.

**Table 4:** Characteristics of formative research participants for the Combination Prevention Effectiveness Study for young cisgender men who have sex with men and transgender women, Thailand (2015-2020).

Characteristics		MSM^a^ (N=41)	TGW^b^ (N=21)	Venue staff (N=24)
Age (years), median (range)		22 (18-26)	24 (20-26)	42 (21-52)
**Completed education, n (%)**				
	Lower than primary	9 (22)	1 (5)	7 (29)
	Primary/secondary	26 (63)	13 (62)	5 (21)
	Vocational	4 (10)	1 (5)	3 (13)
	BA or higher	2 (5)	6 (29)	9 (38)
**Duration exchanging/selling sex, n (%)**				
	<1 month	0 (0)	1 (5)	—^c^
	1-6 months	6 (15)	3 (14)	—
	7-12 months	7 (17)	2 (10)	—
	>12 months	28 (68)	14 (67)	—
	No answer	0 (0)	1 (5)	—
**Location of sex work/exchange, n (%)**				
	Internet only	7 (17)	6 (29)	—
	Venue only	14 (34)	4 (19)	—
	Street-based only	1 (2)	0 (0)	—
	Individual financial support (*Sugar Daddy*)	1 (2)	1 (5)	—
	Combination	18 (44)	10 (48)	—
**Sexual orientation and gender identity (based on local terminology), n (%)**				
	Homosexual, gay cisgender	26 (63)	0 (0)	15 (63)
	Homosexual, transgender	0 (0)	19 (91)	3 (13)
	Heterosexual	8 (20)	0 (0)	4 (17)
	Bisexual	5 (12)	0 (0)	1 (4)
	Do not know/not sure	2 (5)	0 (0)	1 (4)
	Other	0 (0)	2 (10)	0 (0)
**Sexual role, n (%)**				
	Oral	3 (7)	0 (0)	—
	Anal	4 (10)	2 (10)	—
	Both oral and anal	34 (83)	19 (91)	—

^a^MSM: men who have sex with men.

^b^TGW: transgender women.

^c^Not applicable to venue staff as they do not participate in sex work.

### Combination Prevention Intervention

As of February 2019, 445 participants (417 MSM and 28 TGW) have contributed approximately 168 PY with 95% (73/77) retention at 12 months. 74.2% (330/445) of enrolled participants initiated PrEP at baseline, contributing to 134 PY of PrEP adherence based on a measure of 4 or more pills within the last 7 days, 1 PY nonadherence, and 33 PY PrEP nonuse/noninitiation. Monitoring of unexpected events has identified 7 social harms, predominantly related to unintentional participant disclosure of PrEP use and peer stigmatization of PrEP and HIV.

## Discussion

This novel open-label study design and analytic plan will allow evaluation of the effectiveness and cost-effectiveness of combination prevention intervention for people who sell or exchange sex.

The vast majority of cisgender MSM and TGW who exchange sex and participate in this study thus far are interested in PrEP, report taking sufficient PrEP, and stay on PrEP, though additional efforts are needed to address community misinformation and stigma. Given the high interest and uptake of PrEP among young MSM and TGW who exchange sex, we anticipate that one limitation of the study will include the ability to enroll sufficient person-time of non-PrEP use. However, the high uptake, retention, and adherence that has not been observed in other PrEP demonstration projects may provide critical insights into method to improve the current PrEP continuum among MSM and TGW in other settings.

To our knowledge, this is one of the first studies to test the effectiveness of PrEP specifically for MSM and TGW who exchange sex. This study may answer important questions about the effectiveness of PrEP for those in high-risk contexts and the costs of providing such interventions. Furthermore, this study may identify optimal technology-based methods to support PrEP adherence among young populations with high transiency and occupations that would otherwise limit traditional in-person adherence support. In a setting where PrEP has received significant support by the government in HIV prevention and response, estimating the costs and cost-effectiveness of community-based implementation may have important implications for national approaches to bring PrEP to scale in Thailand.

## Appendix

Multimedia Appendix 1 COPE4YMSM NIH summary statement (2015).

## Abbreviations

ACASI: audio computer-assisted self-interview
APCOM: Asia Pacific Coalition on Male Sexual Health
ART: antiretroviral therapy
ARV: antiretroviral
CASI: computer-assisted self-interview
CBO: community-based organization
CDC: Centers for Disease Control and Prevention
COPE: Combination Prevention Effectiveness
DALY: disability-adjusted life year
DBS: dried blood spots
EC: ethical committee
eCrCl: estimated creatinine clearance
FTC: emtricitabine
GDP: gross domestic product
ICER: incremental cost-effectiveness ratio
IPCW: inverse probability of censoring weight
IPTW: inverse probability of treatment weight
IPTCW: inverse probability of treatment and censoring weight
IRB: institutional review board
KII: key informant interview
MOPH: Ministry of Public Health
MSM: men who have sex with men
NIAID: National Institute of Allergy and Infectious Diseases
NAAT: nucleic acid amplification test
NIH: National Institutes of Health
PI: principal investigator
PrEP: pre-exposure prophylaxis
PY: person-years
QC: quality control
RSAT: Rainbow Sky Association of Thailand
SCC: Silom Community Clinic
SEM: Social Ecological Model
STIs: sexually transmitted infections
SWING: Service Workers In Group Foundation
TDF: tenofovir disoproxil fumarate
TGW: transgender women
THB: Thai baht
TUC: Thai-US Collaboration
US CDC: United States Centers for Disease Control and Prevention
