# Supplementary material for: Testing the Effectiveness and Cost-Effectiveness of a Combination HIV Prevention Intervention Among Young Cisgender Men Who Have Sex With Men and Transgender Women Who Sell or Exchange Sex in Thailand: Protocol for the Combination Prevention Effectiveness Study
Source: JMIR Res Protoc. 2020 Jan 27;9(1):e15354. doi: 10.2196/15354 (PMC7011123; doi:10.2196/15354)
Supplement: Multimedia Appendix 1 [file resprot_v9i1e15354_app1.PDF]

SUMMARY STATEMENT
(Privileged Communication)

PROGRAM CONTACT:
Elizabeth Flanagan
240-292-4777
elizabeth.flanagan@nih.gov

Release Date: 04/05/2015

Application Number: 1 R01 AI118505-01A1

Principal Investigator

BEYRER, CHRISTOPHER MD

Applicant Organization: JOHNS HOPKINS UNIVERSITY

Review Group: BSPH
Behavioral and Social Science Approaches to Preventing HIV/AIDS Study Section
AIDS - EXP. REV.

Meeting Date: 03/12/2015
Council: MAY 2015
Requested Start: 07/01/2015
RFA/PA: PA13-302
PCC: A22M

Project Title: Effectiveness of combination HIV preventive interventions for young Thai MSM

SRG Action: Impact Score: 10 Percentile: 2
Next Steps: Visit http://grants.nih.gov/grants/next_steps.htm
Human Subjects: 30-Human subjects involved - Certified, no SRG concerns
Animal Subjects: 10-No live vertebrate animals involved for competing appl.
Gender: 3A-Only men, scientifically acceptable
Minority: 5A-Only foreign subjects, scientifically acceptable
Children: 1A-Both Children and Adults, scientifically acceptable
Clinical Research - not NIH-defined Phase III Trial

Project Year	Direct Costs Requested	Estimated Total Cost
1	450,385	645,804
2	446,556	640,314
3	447,109	641,107
4	447,813	642,116
5	456,757	654,941
TOTAL	2,248,620	3,224,281

ADMINISTRATIVE BUDGET NOTE: The budget shown is the requested budget and has not been adjusted to reflect any recommendations made by reviewers. If an award is planned, the costs will be calculated by Institute grants management staff based on the recommendations outlined below in the COMMITTEE BUDGET RECOMMENDATIONS section.

1R01AI118505-01A1 Beyrer, Christopher

RESUME AND SUMMARY OF DISCUSSION: This outstanding applicant and his very strong collaborators propose testing the efficacy of a combination intervention with and without daily intake of PrEP (Truvada), augmented by mobile phone-based SMS to encourage adherence among young Thai MSM (YMSM) currently engaged in sex work or have done so in the previous 12 months. The high incidence of HIV infection among YMSM and the recorded benefit of PrEP when consistently adhered to support the significance of this application. This initially very strong resubmission has been further strengthened by the team's response to the previous critiques. The study is now well grounded in the Social Ecological Model; in addition, among the many other improvements to the application, concerns about inclusion/exclusion criteria, measures of PrEP adherence, and clarification of the community engagement strategy have been satisfactorily addressed. The potential impact of the proposed study is further increased by the costing and cost effectiveness components proposed as well as by the involvement of important and relevant stakeholders in this research. The latter is not only testimony to this team's experience in the region but also raises confidence in the feasibility of this project. The committee was very enthusiastic about this application which earned its highest level of support.

DESCRIPTION (provided by applicant): This Thai-US collaborative application seeks to develop and conduct a non-randomized effectiveness and cost-effectiveness study of a multi-level combination HIV preventive intervention with and without daily oral Tenofovir/Emtricitabine (Truvada) pre-exposure prophylaxis (PrEP) with mobile phone-based SMS adherence support among young men who have sex with men (YMSM) in Bangkok, Thailand. The current epidemic of HIV among Thai YMSM (men aged 18-26) is marked by high HIV incidence (5-12/100py) despite current prevention and treatment efforts, and rates are highest among the youngest men, aged 18-21, and among the subset of YMSM who sell sex. The proposed combination intervention will include individual level components: frequent HIV testing, risk reduction counseling, condom and lubricant distribution, and behavior change counseling; community awareness and mobilization; and an open label offer of PrEP with SMS (FrontlineSMS) adherence support. Participants will include Thai YMSM engaged in sex work or with a history of recent (in the previous 12 months) selling sex. The proposed study will be conducted by Johns Hopkins, Mahidol, and Emory Universities; The Thai MOPH-US CDC Collaboration (TUC) and its Silom Community Clinic @ Trop Med (SCC); and community partners including SWING, a sex worker empowerment and health promotion group; Rainbow Sky, an LGBT health and rights group; and APCOM, the Asia-Pacific Community of MSM. This group combines unique expertise, access, and experience in working with YMSM in this hyper-epidemic context. The study will include a formative phase using qualitative and community engagement methods to refine the intervention; a pilot; the open label intervention assessing effectiveness of combination interventions with and without PrEP and SMS adherence support with an HIV infection endpoint; and a costing and cost-effectiveness assessment which will measure the costs associated with the combined intervention, the number of infections averted through PrEP use, discounted treatment costs save, and assess whether the intervention packages are cost- saving, cost effective, or not cost-effective. The effectiveness study has been powered on the comparison of person time on PrEP versus not on PrEP and will employ propensity score methods to reduce the potential bias inherent in the self-selection design. In all the study proposes to enroll 1240 YMSM, 620 who choose PrEP and 620 who do not choose PrEP.

PUBLIC HEALTH RELEVANCE: This is a proposal to develop and conduct a non-randomized effectiveness and cost- effectiveness study of a multi-level combination HIV preventive intervention with and without daily oral Truvada pre-exposure prophylaxis (PrEP) with mobile phone-based adherence support among young men who have sex with men (YMSM, aged 18-26) in Bangkok, Thailand. The study will include a formative phase, with the refinement of a multi-level combination intervention; an effectiveness trial with an HIV incidence endpoint; and a costing and cost- effectiveness component among 1240 YMSM in a high HIV incidence context.

CRITIQUE 1:

Significance: 1
Investigator(s): 1
Innovation: 1
Approach: 2
Environment: 1

Overall Impact: The proposed study will assess the effectiveness and cost effectiveness of a combination intervention for prevention of HIV infection among HIV uninfected at risk young (18-26 year old) men who have sex with men (YMSM) in Bangkok, Thailand---a population shown to be at significantly high risk of infection. The combination intervention will include individual level components (regular HIV testing, risk reduction counseling, condom and condom compatible lubricant distribution and behavior change); community awareness and mobilization; and an open label offer of daily oral PrEP with mobile phone-based SMS adherence support. The research team is very strong and inclusive of both US and Thai leadership. The intervention takes an innovative approach to use of PrEP as a targeted intervention component for YMSM transitioning through sex work. The intervention design could perhaps be strengthened by closer consideration of intermediate levels in the social ecological model used to organize it; however, this would need to be balanced against adding further complexity to the design. In summary, the research thoughtfully addresses an urgent need for effective combination prevention that incorporates ARV-based components for YMSM at exceptionally high risk for acquisition and transmission of HIV in Bangkok, regionally in Asia, and globally. As such, it is likely to make a significant contribution to the field of HIV prevention.

1. Significance:

Strengths

- The research addresses a critical need for effective combination prevention in a key population (YMSM engaged in sex work) locally, regionally and globally.

Weaknesses

- None noted.

2. Investigator(s):

Strengths

- The PI, Dr. Beyrer, has an impressive record of research that is highly relevant to the proposed work.
- The site PI, Dr. Holtz, has a similarly impressive track record and is currently directing the Thai MOPH/CDC collaboration in Bangkok.
- The study statistician, Dr. Rose, served in a similar role for the CDC Botswana PrEP trial and contributed to analysis of the Bangkok Tenofovir Study.
- Other key persons at JHU, CDC, Emory University and Mahidol University bring comprehensive expertise in support of the proposed research including social epidemiology, medical anthropology, vulnerable populations, including adolescents and sex workers, participatory research, comprehensive prevention for MSM, adherence, and HIV testing.

Weaknesses

- Dr. Decker's contributions are described in her biosketch as providing guidance on the development, execution and evaluation of the community empowerment component of the combination intervention. However, she is not included in the otherwise comprehensive listing of collaborators in the research strategy.
- Biosketch for one of the key Thai collaborators is missing (Dr. Pachara, proposed as co-investigator and member of the study Executive Committee).

3. Innovation:

Strengths

- The intervention design is an innovative approach to addressing the needs of a dynamic at-risk population (YMSM transitioning in and out of sex work).
- The intervention brings together elements that have evidence of effectiveness and address HIV risk, including behavioral, biomedical, and community-based elements.

Weaknesses

- None noted.

4. Approach:

Strengths

- The effectiveness assessment includes a clearly stated null hypothesis.
- Discontinuation of PrEP---an important component for the effectiveness assessment---has been defined/operationalized as follows: "Participants will be categorized as discontinued and off PrEP on the day after their last dose if they report either not taking PrEP for more than 30 days or not taking PrEP for less than 30 days but report a desire to discontinue PrEP use. Medication pick-ups will be used to verify cessation and/or (re)initiation of PrEP use."
- Previous concerns about lack of PrEP adherence measurement have been addressed through use of 3 measures (self-report adapted from an ACTG instrument, monthly medication pick up, and use of dried blood spots to assess Tenofovir diphosphate levels).
- Neutral Assessment will be used to assess adherence to prevention packages broadly and use of PrEP specifically.
- Recruitment criteria have been clarified and are appropriate (MSM aged 18-26 who report current or past (within the previous 12 months) history of having sold or exchanged sex with other men for money, drugs, or other goods).
- The qualitative component is now more closely targeted to refining the intervention design and procedures.
- The objectives of the pilot study are also now more targeted and criteria for inclusion of pilot participants in the total sample set out. The pilot timeline (12 weeks) allows for integration of findings to the intervention design.
- The community engagement strategy is now fully described and includes plans for an evaluation of the community mobilization efforts using resources from the Community Tool Box (developed by Stephen Fawcett).
- The relationship between the community engagement strategy and ethical considerations in use of PrEP for men who sell sex has been clarified.

- The joint use of FrontlineSMS and Next Step Counseling to promote adherence is a strength.
- The relationship between the study outcomes and elements of the intervention have been clarified, and aligned with the Social Ecological Model being used to inform the intervention design.

Weaknesses

- The timing of the collection of the dried blood spots for assessing PrEP adherence appears to be monthly (i.e., at monthly study visits), but is not explicitly specified.
- Reference is made to “Figure one shows the levels of influence which have been shown to impact PrEP uptake and adherence among MSM, including one study of Thai MSM in the IPrEX OLE.” However, the actual Figure 1 is a project management plan and study timeline. It appears the reference is meant to be to Fig 2, the Social Ecological Model. This was confusing, but more importantly the associated text indicates evidence that family support “was found to be important in PrEP adherence in a recent Thai study” but the proposed intervention does not explicitly address this level of the model or give a rationale for not addressing this level. The exercise of including the SEM conceptual framework seems to have helped organize aspects of the intervention design, but the design elements appear to have been minimally informed by the framework.

5. Environment:

Strengths

- The collaborating organizations -- Johns Hopkins, Mahidol, and Emory Universities; The Thai MOPH-US CDC Collaboration (TUC) and its Silom Community Clinic @ Trop Med (SCC); and community partners including SWING, a sex worker empowerment and health promotion group; Rainbow Sky, an LGBT health and rights group; and APCOM, the Asia-Pacific Community of MSM – provide an exceptionally strong environment for the proposed research.

Weaknesses

- None noted.

Protections for Human Subjects:

Acceptable Risks and/or Adequate Protections

- Risks and protections are comprehensively described and are acceptable/adequate.

Data and Safety Monitoring Plan (Applicable for Clinical Trials Only):

Acceptable

- A Safety Monitoring Committee (SMC) will be established to review study progress, assess any social harms, review adverse events reported by the site PI, and specify safety and effectiveness stopping rules. This is comprehensively described.

Inclusion of Women, Minorities and Children and not IRB Exemption #4.

- Sex/Gender: Distribution justified scientifically
- Race/Ethnicity: Distribution justified scientifically
- Inclusion/Exclusion of Children under 21: Including ages < 21 justified scientifically

- In Thailand, persons aged 18-21 have attained the legal age of consent for HIV/STI services, including HIV testing. Women are excluded from the study because the study aims relate to developing and testing HIV prevention packages for men who have sex with men.

Vertebrate Animals:

Not Applicable (No Vertebrate Animals)

Biohazards:

Not Applicable (No Biohazards)

Resubmission:

- The investigators responded to all of the concerns raised in the previous review, strengthening an already strong proposal.

Applications from Foreign Organizations:

Justified

- The proposed research addresses a priority population for global HIV prevention (young Thai MSM).

Resource Sharing Plans:

Acceptable

Budget and Period of Support:

Recommend as Requested:

- Clarification of Dr. Decker's role in the study in year 1 is needed.

CRITIQUE 2:

Significance: 1
Investigator(s): 1
Innovation: 1
Approach: 2
Environment: 1

Overall Impact: The revised application proposes a trial of a combination prevention intervention with or without PrEP for young men who have sex with men in Thailand. The PI and the rest of the investigator team are strong with excellent support from their respective institutions. The study design and strategies are well developed to answer the proposed research questions. Analytic plans are appropriate. Special attention to ethical considerations in use of PrEP for men who sell sex is commendable.

1. Significance:

Strengths

- To develop and assess the effectiveness of a combination prevention with or without PrEP is highly significant.
- To address HIV prevention for YMSM sex workers in Thailand is critical.

Weaknesses

- None Noted

2. Investigator(s):

Strengths

- The Thai-US collaborative team is strong and well qualified to carry out the study.

Weaknesses

- None Noted

3. Innovation:

Strengths

- Multiple methods will be used to measure PrEP adherence.
- The inclusion of a community mobilization component is innovative.

Weaknesses

- None Noted

4. Approach:

Strengths

- Intervention procedures will be piloted before the trial.
- Mobile/SMS support for adherence.
- The proposed cost analysis plan is well thought out.

Weaknesses

- Seven follow-ups with HIV testing within 12 months could be a big burden to participants.

5. Environment:

Strengths

- The environment and support are excellent.

Weaknesses

- None noted

Protections for Human Subjects:

Acceptable Risks and/or Adequate Protections

Data and Safety Monitoring Plan (Applicable for Clinical Trials Only):

Acceptable

Inclusion of Women, Minorities and Children and not IRB Exemption #4.

- Sex/Gender: Distribution justified scientifically
- Race/Ethnicity: Distribution justified scientifically
- Inclusion/Exclusion of Children under 21: Including ages < 21 justified scientifically

Vertebrate Animals:

Not Applicable (No Vertebrate Animals)

Biohazards:

Not Applicable (No Biohazards)

Applications from Foreign Organizations:

Justified

Resource Sharing Plans:

Acceptable

Budget and Period of Support:

Recommend as Requested:

CRITIQUE 3:

Significance: 1
Investigator(s): 1
Innovation: 1
Approach: 2
Environment: 1

Overall Impact: This revised application proposes a non-randomized comparative 2-arm trial of combination HIV prevention with and without PrEP for YMSM sex workers in Thailand. As noted in the prior review, there were various strengths; these are retained in this revision. Significance includes focus on a population at extreme risk, building on the results of 4 promising RCTs to address behavioral uptake in real world settings. The application uses innovative, methodologically sound design, by a stellar team situated superbly. The prior review identified four main weaknesses which this revision has addressed. High potential impact.

1. Significance:

Strengths

- As noted in the prior review, the population under study is at critical HIV risk, the research questions are informed by the success of 4 RCTs of PrEP but have potential to advance the field on the chief challenge of adherence, and the approach emphasizes “real world” implementation.
- The prior review noted the omission of a theoretical model. In this revision, the applicant has added the modified-SEM model (fortuitously recently published by this team). The model appears appropriate to the study questions and further shows the depth of reflection this team has undertaken integrating structural and network factors into one model.

Weaknesses

- None Noted

2. Investigator(s):

Strengths

- As noted in prior review, the investigators are a very strong; they are led by a pre-eminent researcher in this area and supported by an outstanding team in all areas.
- The prior review noted the biosketches of some investigators omitted role on study.

Weaknesses

- None Noted

3. Innovation:

Strengths

- As noted on prior review, research design, study of cross-over effects of PrEP uptake and adherence, real world impacts in this population, comprehensive approach, and community consultation all contribute to a highly innovative study.

Weaknesses

- None Noted

4. Approach:

Strengths

- As noted in prior review, the overall approach, methods, measures, data collection, and analysis is sound.
- A weakness noted in prior review was loose subject eligibility criterion. This revision now restricts eligibility to MSM with recent evidence of sex work which addresses this concern.
- There was a concern about adherence measures. The applicant has clarified they will be using CDC-PrEP counseling measures as operationalized in the ACTG instrument, and cross-validated with monthly medication pick up rates and biomeasures. This appears state-of-practice (and research).
- In response for more detail about the intervention approach the applicant has added detail at each level of the mod-SEM model.

Weaknesses

- None Noted

5. Environment:

Strengths

- As noted in prior review, the academic environment, relationship to community, and real world setting for this study are stellar and uniquely positioned to advance research on PrEP.
- The Bangkok setting adds credibility for pan-Asian uptake if found effective.

Weaknesses

- None Noted

Protections for Human Subjects:

Unacceptable Risks and/or Inadequate Protections

- As noted in prior review protections against medical risks related to PrEP are not described. Specifically it is unclear whether and what side effects will be monitored.

Data and Safety Monitoring Plan (Applicable for Clinical Trials Only):

Unacceptable

- The prior review noted the absence of a DSMP. In this review, the applicant has identified a DSMB, but states that a DSMP "will be written and agreement about the plan reached with the SMC." While this has the advantage of not burdening external colleagues in a developing world context with work prior to determining success of the application, it prevents reviewers from examining the specifics (and/or offering helpful suggestions). The elements listed by the PI clearly show familiarity with all elements of a DSMP and his prior experience in running biobehavioral trials adds confidence this requirement will be addressed. Nevertheless, the approach of planning the plan rather than detailing it is less than optimal for review.

Inclusion of Women, Minorities and Children and not IRB Exemption #4.

- Sex/Gender: Distribution justified scientifically
- Race/Ethnicity: Distribution justified scientifically
- Inclusion/Exclusion of Children under 21: Including ages < 21 justified scientifically
- In prior review, the applicant received feedback to consider whether the lower age of 18 was scientifically justified given evidence of early sexual debut. In response, the applicant argues that given Thai policies restricting sex work and HIV services to those age 18+, lowering the age limit is problematic. Responsive

Vertebrate Animals:

Not Applicable (No Vertebrate Animals)

Biohazards:

Not Applicable (No Biohazards)

Resubmission:

- With one exception, the applicant has been highly responsive to the prior review. The absence of a written DSMP, which covers monitoring of side effects, remains an unfulfilled requirement for this application.

Applications from Foreign Organizations:

Justified

- The inclusion of investigators from the Thailand Ministry of Health and Mahidol University, together with the siting of the study in Bangkok is supported by strong scientific and epidemiologic justification.

Budget and Period of Support:

Recommend as Requested:

THE FOLLOWING SECTIONS WERE PREPARED BY THE SCIENTIFIC REVIEW OFFICER TO SUMMARIZE THE OUTCOME OF DISCUSSIONS OF THE REVIEW COMMITTEE, OR REVIEWER'S WRITTEN CRITIQUES, ON THE FOLLOWING ISSUES:

PROTECTION OF HUMAN SUBJECTS: ACCEPTABLE

INCLUSION OF WOMEN PLAN (G3A): ACCEPTABLE

INCLUSION OF MINORITIES PLAN (M5A): ACCEPTABLE

INCLUSION OF CHILDREN PLAN (C1A): ACCEPTABLE

COMMITTEE BUDGET RECOMMENDATIONS: The budget was recommended as requested.

NIH has modified its policy regarding the receipt of resubmissions (amended applications). See Guide Notice NOT-OD-14-074 at http://grants.nih.gov/grants/guide/notice-files/NOT-OD-14-074.html. The impact/priority score is calculated after discussion of an application by averaging the overall scores (1-9) given by all voting reviewers on the committee and multiplying by 10. The criterion scores are submitted prior to the meeting by the individual reviewers assigned to an application, and are not discussed specifically at the review meeting or calculated into the overall impact score. Some applications also receive a percentile ranking. For details on the review process, see http://grants.nih.gov/grants/peer_review_process.htm#scoring.

MEETING ROSTER

Behavioral and Social Science Approaches to Preventing HIV/AIDS Study Section
AIDS and Related Research Integrated Review Group
CENTER FOR SCIENTIFIC REVIEW
BSPH
March 12, 2015 - March 13, 2015

CHAIRPERSON

FLYNN, PATRICK M, PHD
PROFESSOR AND DIRECTOR
INSTITUTE OF BEHAVIORAL RESEARCH
DEPARTMENT OF PSYCHOLOGY
TEXAS CHRISTIAN UNIVERSITY
FT WORTH, TX 76109

MEMBERS

ALLEN, SUSAN A, MPH, MD
PROFESSOR
DEPARTMENT OF PATHOLOGY
AND LABORATORY MEDICINE
EMORY UNIVERSITY
ATLANTA, GA 30322

BANKOLE, AKINRINOLA , PHD
DIRECTOR
INTERNATIONAL RESEARCH
THE GUTTMACHER INSTITUTE
NEW YORK, NY 10005

BLANKENSHIP, KIM M, PHD
PROFESSOR AND CHAIR
DEPARTMENT OF SOCIOLOGY
AMERICAN UNIVERSITY
WASHINGTON, DC 20016

BOEKELOO, BRADLEY O, PHD *
PROFESSOR
DEPARTMENT OF BEHAVIORAL AND COMMUNITY
HEALTH
SCHOOL OF PUBLIC HEALTH
UNIVERSITY OF MARYLAND
COLLEGE PARK, MD 20742

BOURGOIS, PHILIPPE , PHD
PROFESSOR
DEPARTMENTS OF ANTHROPOLOGY
AND FAMILY AND COMMUNITY MEDICINE
UNIVERSITY OF PENNSYLVANIA
PHILADELPHIA, PA 19104

BROWN, LARRY K, MD *
PROFESSOR
DEPARTMENT OF PSYCHIATRY AND HUMAN BEHAVIOR
SCHOOL OF MEDICINE
BROWN UNIVERSITY
PROVIDENCE, RI 02912

CHARLEBOIS, EDWIN DUNCAN III, MPH, PHD
PROFESSOR
DEPARTMENT OF MEDICINE
SCHOOL OF MEDICINE
UNIVERSITY OF CALFORNIA, SAN FRANCISCO
SAN FRANCISCO , CA 94105

COATES, THOMAS J, PHD *
PROFESSOR AND DIRECTOR
DEPARTMENT OF MEDICINE
PROGRAM OF GLOBAL HEALTH
DAVID GEFFEN SCHOOL OF MEDICINE
UNIVERSITY OF CALIFORNIA, LOS ANGELES
LOS ANGELES, CA 90024

DANGERFIELD, BRIAN CHARLES, PHD *
PROFESSOR
DEPARTMENT OF MANAGEMENT
SCHOOL OF ECONOMICS, FINANCE AND MANAGEMENT
UNIVERSITY OF BRISTOL
BRISTOL, BS8 1TN
UNITED KINGDOM

ELDRIDGE, GLORIA D, PHD *
ASSOCIATE PROFESSOR
DEPARTMENT OF PSYCHOLOGY
UNIVERSITY OF ALASKA ANCHORAGE
ANCHORAGE, AK 99508

ENAH, COMFORT CHU, PHD, BSN
ASSISTANT PROFESSOR
COMMUNITY HEALTH OUTCOMES
AND SYSTEMS DEPARTMENT
SCHOOL OF NURSING
UNIVERSITY OF ALABAMA AT BIRMINGHAM
BIRMINGHAM, AL 35294

ESSIEN, EKERE JAMES, MD, DRPH *
PROFESSOR
DEPARTMENT OF CLINICAL SCIENCES
AND ADMINISTRATION
COLLEGE OF PHARMACY
UNIVERSITY OF HOUSTON
HOUSTON, TX 77030

FEASTER, DANIEL J, PHD
ASSOCIATE PROFESSOR
DIVISION OF BIOSTATISTICS
DEPARTMENT OF PUBLIC HEALTH SCIENCES
UNIVERSITY OF MIAMI
MIAMI, FL 33136

GOGGIN, KATHY J, PHD *
PROFESSOR
DEPARTMENT OF PSYCHOLOGY
UNIVERSITY OF MISSOURI, KANSAS CITY
KANSAS CITY, MO 64110

HAVENS, JENNIFER R, MPH, PHD
ASSOCIATE PROFESSOR
DEPARTMENT OF BEHAVIORAL SCIENCE
COLLEGE OF MEDICINE
UNIVERSITY OF KENTUCKY
LEXINGTON, KY 40504

HECKMAN, TIMOTHY GLENN, PHD
PROFESSOR
HEALTH PROMOTION AND BEHAVIOR
COLLEGE OF PUBLIC HEALTH
UNIVERSITY OF GEORGIA
ATHENS, GA 30602

HIGHTOW-WEIDMAN, LISA B, MD *
CLINICAL ASSOCIATE PROFESSOR
DIVISION OF INFECTIOUS DISEASES
DEPARTMENT OF MEDICINE
UNIVERSITY OF NORTH CAROLINA SCHOOL OF MEDICINE
CHAPEL HILL, NC 275997030

KURTZ, STEVEN P, PHD
PROFESSOR AND DIRECTOR
CENTER FOR APPLIED RESEARCH ON SUBSTANCE USE AND HEALTH DISPARITIES
NOVA SOUTHEASTERN UNIVERSITY
CORAL GABLES, FL 33134

LEVY, JUDITH A, PHD *
DIRECTOR
FOGARTY AIDS INTERNATIONAL
TRAINING AND RESEARCH PROGRAM
UNIVERSITY OF ILLINOIS AT CHICAGO
CHICAGO, IL 60612

LI, LI , PHD
PROFESSOR
DEPARTMENT OF PSYCHIATRY AND BEHAVIORAL SCIENCES
UNIVERSITY OF CALIFORNIA, LOS ANGELES
LOS ANGELES, CA 90024

MACQUEEN, KATHLEEN M, PHD
SENIOR SOCIAL SCIENTIST
BEHAVIORAL AND SOCIAL SCIENCES
FAMILY HEALTH INTERNATIONAL
UNIVERSITY OF NORTH CAROLINA AT CHAPEL HILL
DURHAM, NC 27713

MARTINEZ-DONATE, ANA P, PHD
ASSOCIATE PROFESSOR
DEPARTMENT OF POPULATION HEALTH SCIENCES
UNIVERSITY OF WISCONSIN
MADISON, WI 53726

MORRIS, WANDA MARTINA, PHD *
PROFESSOR
DEPARTMENTS OF SOCIOLOGY AND STATISTICS
UNIVERSITY OF WASHINGTON
SEATTLE, WA 98195

O'DONNELL, LYDIA N, EDD *
DIRECTOR
HEALTH AND HUMAN DEVELOPMENT DIVISION
EDUCATION DEVELOPMENT CENTER INCORPORATED
NEWTON, MA 02458

ROSSER, B R SIMON , MPH, PHD
PROFESSOR AND DIRECTOR
DIVISION OF EPIDEMIOLOGY AND COMMUNITY HEALTH
UNIVERSITY OF MINNESOTA
MINNEAPOLIS, MN 55454

SIMONI, JANE MARIE, PHD
PROFESSOR
DEPARTMENT OF PSYCHOLOGY
UNIVERSITY OF WASHINGTON
SEATTLE, WA 98195

SLESNICK, NATASHA , PHD *
PROFESSOR
DEPARTMENT OF HUMAN DEVELOPMENT AND FAMILY SCIENCE
OHIO STATE UNIVERSITY
COLUMBUS, OH 43210

WEINHARDT, LANCE S, PHD
PROFESSOR
DEPARTMENT OF PSYCHIATRY
JOSEPH J ZILBER SCHOOL OF PUBLIC HEALTH
UNIVERSITY OF WISCONSIN, MILWAUKEE
MILWAUKEE, WI 53201

WENZEL, SUZANNE L, PHD
PROFESSOR
SCHOOL OF SOCIAL WORK
UNIVERSITY OF SOUTHERN CALIFORNIA
LOS ANGELES, CA 90089

MAIL REVIEWER(S)

STEWART, KATHARINE E, MPH, PHD
ASSOCIATE PROFESSOR AND ASSOCIATE DEAN
DEPARTMENT OF HEALTH BEHAVIOR AND HEALTH EDUCATION
COLLEGE OF PUBLIC HEALTH
UNIVERSITY OF ARKANSAS FOR MEDICAL SCIENCES
LITTLE ROCK, AR 72205

WOOD, EVAN , MD, PHD
PROFESSOR
CENTRE FOR EXCELLENCE IN HIV/AIDS
ST PAUL HOSPITAL
BRITISH COLUMBIA UNIVERSITY
VANCOUVER, BC V6Z 1Y6
CANADA

SCIENTIFIC REVIEW OFFICER

GUERRIER, JOSE H, PHD
SCIENTIFIC REVIEW OFFICER
CENTER FOR SCIENTIFIC REVIEW
NATIONAL INSTITUTES OF HEALTH
BETHESDA, MD 20892

EXTRAMURAL SUPPORT ASSISTANT

STROTHERS, DIARA
EXTRAMURAL SUPPORT ASSISTANT
CENTER FOR SCIENTIFIC REVIEW
NATIONAL INSTITUTES OF HEALTH
BETHESDA, MD 20892

* Temporary Member. For grant applications, temporary members may participate in the entire meeting or may review only selected applications as needed.

Consultants are required to absent themselves from the room during the review of any application if their presence would constitute or appear to constitute a conflict of interest.
